# Assessing Conifer Ray Parenchyma for Ecological Studies: Pitfalls and Guidelines

**DOI:** 10.3389/fpls.2015.01016

**Published:** 2015-11-18

**Authors:** Georg von Arx, Alberto Arzac, José M. Olano, Patrick Fonti

**Affiliations:** ^1^Landscape Dynamics Research Unit, Swiss Federal Institute for Forest, Snow and Landscape Research WSLBirmensdorf, Switzerland; ^2^Departamento de Biología Vegetal y Ecología, Facultad de Ciencia y Tecnología, Universidad del País VascoLeioa, Spain; ^3^Departamento de Ciencias Agroforestales, Escuela Universitaria de Ingenierías Agrarias, Instituto Universitario de Investigación en Gestión Forestal Sostenible-Universidad de ValladolidSoria, Spain

**Keywords:** cutting plane, measured wood surface, measurement accuracy, non-structural carbohydrates (NSC), number of samples, ray density, ray dimensions, ray volume

## Abstract

Ray parenchyma is an essential tissue for tree functioning and survival. This living tissue plays a major role for storage and transport of water, nutrients, and non-structural carbohydrates (NSC), thus regulating xylem hydraulics and growth. However, despite the importance of rays for tree carbon and water relations, methodological challenges hamper knowledge about ray intra- and inter-tree variability and its ecological meaning. In this study we provide a methodological toolbox for soundly quantifying spatial and temporal variability of different ray features. Anatomical ray features were surveyed in different cutting planes (cross-sectional, tangential, and radial) using quantitative image analysis on stem-wood micro-sections sampled from 41 mature Scots pines (*Pinus sylvestris*). The percentage of ray surface (PERPAR), a proxy for ray volume, was compared among cutting planes and between early- and latewood to assess measurement-induced variability. Different tangential ray metrics were correlated to assess their similarities. The accuracy of cross-sectional and tangential measurements for PERPAR estimates as a function of number of samples and the measured wood surface was assessed using bootstrapping statistical technique. Tangential sections offered the best 3D insight of ray integration into the xylem and provided the most accurate estimates of PERPAR, with 10 samples of 4 mm^2^ showing an estimate within ±6.0% of the true mean PERPAR (relative 95% confidence interval, CI95), and 20 samples of 4 mm^2^ showing a CI95 of ±4.3%. Cross-sections were most efficient for establishment of time series, and facilitated comparisons with other widely used xylem anatomical features. Earlywood had significantly lower PERPAR (5.77 vs. 6.18%) and marginally fewer initiating rays than latewood. In comparison to tangential sections, PERPAR was systematically overestimated (6.50 vs. 4.92%) and required approximately twice the sample area for similar accuracy. Radial cuttings provided the least accurate PERPAR estimates. This evaluation of ray parenchyma in conifers and the presented guidelines regarding data accuracy as a function of measured wood surface and number of samples represent an important methodological reference for ray quantification, which will ultimately improve the understanding of the fundamental role of ray parenchyma tissue for the performance and survival of trees growing in stressed environments.

## Introduction

Parenchyma in the xylem is a neglected living tissue in ecological and eco-physiological research, despite its essential role for tree carbon and water relations and thus tree survival in response to environmental stochasticity. More specifically, it is important for buffering temporal imbalances between carbon uptake and consumption (McDowell and Sevanto, 2010), and has been principally documented to be involved in the storage and transport of water and nutrients (Witt and Sauter, 1994; Gartner et al., 2000). Additionally, parenchyma provides carbohydrates and water for refilling embolized conduits (Salleo et al., 2009; Brodersen and Mcelrone, 2013; Spicer, 2014), is involved in the formation of heartwood (Bamber, 1976), the defense against pathogens (Hudgins et al., 2006), in wounding processes (Arbellay et al., 2010, 2012), and contributes to the mechanical strength of the wood (Burgert and Eckstein, 2001; Fonti and Frey, 2002).

Due to this important role for tree functioning, pioneer studies on parenchyma mainly focused on its anatomical characterization and quantification among different species (e.g., Myer, 1922; Bannan, 1937; Brown et al., 1949). It has been observed that ray parenchyma represents 3–12% and 5–42% of the overall stem xylem tissue in conifers and angiosperms, respectively (Myer, 1922; Panshin and De Zeeuw, 1980; Brandes and Barros, 2008), making ray parenchyma by far the most important living tissue in the xylem despite substantial contribution of axial parenchyma in some angiosperm species (Spicer, 2014). Parenchyma in the stem sapwood stores a large proportion (25–40%) of the overall non-structural carbohydrates (NSC) reserves of a tree, which makes it a more important NSC reservoir than the phloem or the leaves (Jacquet et al., 2014).

Recently, triggered by the increasing awareness of global change impact on forest ecosystems and tree mortality (Allen et al., 2010; Choat et al., 2012; Lloret et al., 2013; Hereş et al., 2014), there is a renewed and increased need for a better ecological understanding of the role of parenchyma (mainly rays) for tree performance and survival in response to environmental variability (e.g., Olano et al., 2013; Fonti et al., 2015), and particularly with respect to carbon and water balance in stressed trees (Pruyn et al., 2005; Salleo et al., 2006; Esteban et al., 2012; Barnard et al., 2013; Gruber et al., 2013; Rosell et al., 2014). However, the understanding of the ecological role of this tissue has been hampered, mainly due to the scarce data about how parenchyma varies along ecological gradients and/or during tree life, and due to the diversity of methods used for quantification, making comparisons difficult. A review of the measured parameters used for ecological investigations is summarized in Table 1. In general, ray properties have been quantified either in terms of “amount” as proxy for investigating variability in tree vigor, growth conditions and storage capacity; and in terms of “spatial distribution” to explore the integration of rays in the 3D xylem network, which is key for its function as storage and transport tissue connecting the xylem and phloem (Spicer, 2014). These observations seem to reveal that within-tree variability is relatively small compared to the variability observed among individuals (Wimmer and Grabner, 2000; Olano et al., 2013; Fonti et al., 2015). Moreover, it has been observed that ray features co-vary at within-tree level related to organ and age, thus reflecting functional needs and/or allometric scaling in relation to variation in conduit size (Carlquist, 1982; Lev-Yadun and Aloni, 1995; Fonti et al., 2015). An open question in this regard is whether ray characteristics might also show some intra-annual variability. Overall, the observed variations suggest a rather strong intrinsic control of the ray characteristics (Aloni, 2013), leaving less room for identifying plastic responses to external environmental factors. Nevertheless, higher percentage of ray tissue appears to be related to tree performance (Table 1); and annual time series of anatomical features such as the number of initiating rays and the percentage of ray tissue to respond to environmental conditions (Eckstein, 2013; Olano et al., 2013).

**Table 1:** **Literature review of measured anatomical ray parameters and the inferred ecological interpretation of their variability**.

**Ray parameter**	Plane^*^	**Documented and hypothesized variability and potential functional meaning**
**AMOUNT AND DIMENSIONS: RELATED TO TREE VIGOR, GROWTH CONDITIONS, MAXIMUM STORAGE CAPACITY**		
Percentage of ray surface (PERPAR)	C, T, R	-Increase in individuals with higher growth rate (Harlow, 1927; Bannan, 1954, 1965; Gregory, 1977; Fonti et al., 2015), dominant individuals (Myer, 1922), lowland individuals (Myer, 1922), fully illuminated individuals (Hartig, 1894), individuals with larger leaf area (Gartner et al., 2000), with increasing aridity (Esteban et al., 2010, 2012; von Arx et al., 2012), after wounding (Lev-Yadun and Aloni, 1992; Arbellay et al., 2010, 2012) -Responses to short-term climate variability (Olano et al., 2013)
Number of continuing rays (in time series)	C, R	-Responses to short-term climate variability (Olano et al., 2013)
Number of initiating rays (in time series)	C, R	-Responses to short-term climate variability (Olano et al., 2013)
Ray height in metric units	T, R	- Increase with conduit size (Carlquist, 1982; Lev-Yadun and Aloni, 1995; Fonti et al., 2015), distance from pith (DeSmidt, 1922; Lev-Yadun and Aloni, 1995; Falcon-Lang, 2005; Aloni, 2013), with increasing growth rate (Bannan, 1965; Lev-Yadun, 1998; but see White and Robards, 1966), also at same distance from pith (Bannan, 1937) - Decreased close to wounding (Lev-Yadun and Aloni, 1992) - Responses to short-term climate variability (Wimmer and Grabner, 2000), environmental stress (Wimmer, 2002)
Ray height in cell counts	T, R	- Increased from pith to bark (Weinstein, 1926; Gregory, 1977; Lev-Yadun and Aloni, 1992), with growth rate (Bannan, 1965; Gregory and Romberger, 1975)
Ray width in metric units	T	- Increase with growth rate (White and Robards, 1966; Wimmer and Grabner, 2000) - Decreased after wounding (Lev-Yadun and Aloni, 1992)
Ray cell size	T, R	- Increased after wounding (Lev-Yadun and Aloni, 1992)
**SPATIAL DISTRIBUTION: FUNCTIONAL RAY INTEGRATION IN THE 3D XYLEM NETWORK, SEASONAL CHANGES IN STORAGE SPACE REQUIREMENTS**		
Ray density (No·mm^−2^)	C, T, R	- Increase from bark to pith (Lev-Yadun, 1998; Falcon-Lang, 2005), in branches compared to stems (Jaccard, 1915), with higher growth rate [(White and Robards, 1966; Fonti et al., 2015); but see (Lev-Yadun, 1998)], after wounding (Lev-Yadun and Aloni, 1992), with increasing aridity (Esteban et al., 2012) - Reduced due to air pollution (Von Schneider and Halbwachs, 1989)
Ray cell density (No·mm^−2^)	T	- Responses to short-term climate variability (Wimmer and Grabner, 2000) - Increase with aridity (Esteban et al., 2012)
Position of initiating rays (in time series)	C, R	- Preferential initiation of rays in latewood to meet seasonal changes in storage space requirements?

* *Parameter accessible in: C, cross-section; T, tangential section; R, radial section*.

**Figure 1:**
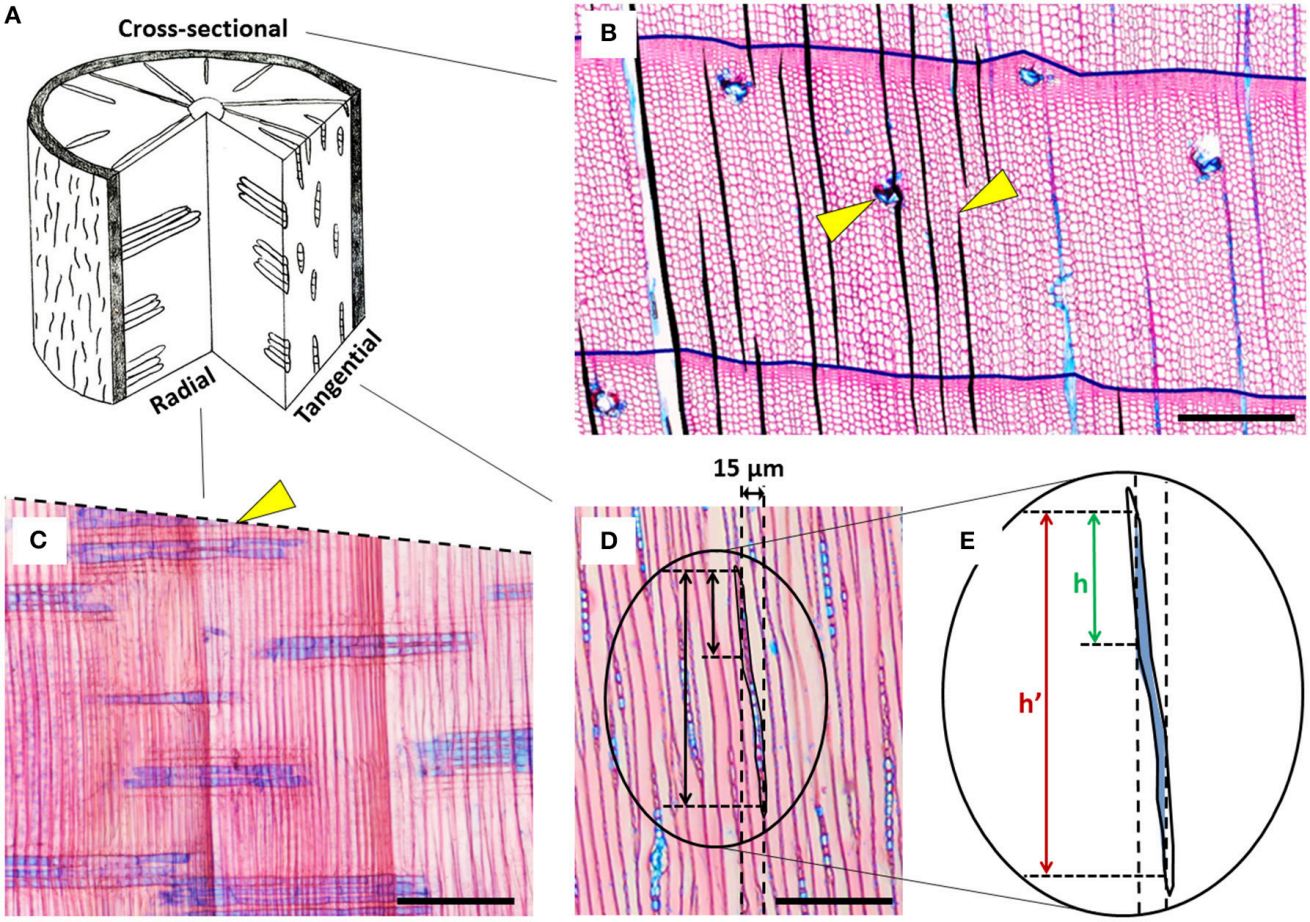
**Overview of the different cutting planes and exemplary anatomical cut-out images of ***Pinus sylvestris*** with rays stained in blue. (A)** Scheme of the different cutting planes in a stem disc showing xylem rays in a 3D wood context. **(B)** Cross-section showing some exemplary rays (area filled black) and tree-ring borders (black lines); the right arrow indicates a disappearing ray, likely due to non-perpendicular orientation of the section; the left arrow indicates a ray with an incorporated resin duct that was excluded from analysis. **(C)** Radial section; the dashed line indicates the non-perpendicular orientation of the cross-section, the arrow points at an example of the “ending ray artifact” in the cross-sectional plane, i.e., permanently disappearing rays. The seemingly short rays show that the core was not in parallel to the radial wood structure. **(D)** Tangential section; the vertical dashed lines simulate a 15 μm thick radial section and the associated “radial overestimation artifact.” **(E)** Zoomed-in sketch of the “radial overestimation artifact” demonstrating that the actual height h is much shorter than the measured height h'. Because of the transparency of the tissue, the perceived ray contour corresponds to the maximum ray projection through the entire thickness of the section. Horizontal black bars in all anatomical images indicate 100 μm.

Disentangling the comparably weak environmental signal from the strong intrinsic and allometric component of ray variability requires both a mechanistic understanding of the processes triggering ray formation, and an appropriate methodological toolbox for a sufficiently accurate quantification. However, the relatively few studies performed so far were using different metrics (e.g., ray area, density, size) quantified on different wood cutting planes (i.e., tangential, cross-sectional, or radial planes; see Figure 1) of varying measured wood surface, thus hampering comparisons among studies. Consequently, many questions as to the best practice remain unanswered. The studies involving parenchyma quantification have mostly used tangential sections, in which typically a surface of just about 1 mm^2^ per sample was measured (e.g., Pruyn et al., 2005; Esteban et al., 2010, 2012), but sometimes surfaces up to 4 mm^2^ were also considered (Fonti et al., 2015). Few ecological studies—for practical reasons when building up time-series along tree rings—have also quantified rays in cross-sections from 5-mm increment cores (Olano et al., 2013; Arzac, 2014; Fonti et al., 2015), whereas radial sections have hardly been, if at all, used for ray quantification.

In this study we aimed at clarifying some methodological issues related to the quantification of ray tissue in conifer wood and giving some guidelines for ecological studies. In particular we focused on the influence of the methodological approach on the quantification of ray features by comparing several ray metrics in (i) different cutting planes, (ii) within the annual ring (earlywood vs. latewood), and (iii) as a function of the measured wood surface. These aspects were addressed using *Pinus sylvestris* L. as a model conifer species.

## Materials and methods

### Study material

*Pinus sylvestris* (Scots Pine) is a sub-boreal evergreen conifer with one of the largest distribution ranging between Scotland and northeast Asia (Nikolov and Helmisaari, 1992) at altitudes between 0 to 2700 m asl. As most conifers, it contains uniseriate rays characterized by a single layer of parenchyma cells (Lev-Yadun and Aloni, 1995; Figure 1) facultatively embraced by tracheid cells on the upper and lower extremities, and sometimes enclosing resin ducts (Brown et al., 1949).

Wood samples for this study were obtained in May 2013 in the xeric Pfynwald forest located in the Swiss Rhone Valley (Valais). The climate at this site is continental with 600–700 mm of annual precipitation and a mean annual temperature of 10.1°C (data from 1981 to 2010 of the weather station of Sion, at 20 km distance from the site, MeteoSwiss). Cores with a diameter of 10 mm were taken at stem breast height from 40 mature Scots pines of 10–13 m height, 12–30 cm dbh, and aged between 45 and 135 years. Ring widths in the cores were measured using a LinTab device and time series were cross-dated using COFECHA (Grissino-Mayer, 2001) to assign each ring to the correct calendar year. The 20 outmost annual rings from all the 40 cores were considered for cross-sectional analyses, whereas a subset of six cores was used for additional tangential and radial analyses at three locations each separated by five to seven annual rings. In addition, a single stem disc of 14 cm diameter (58 years old) was collected from a felled individual, split in three radial bars of 20 mm width and 20 mm height at offsets of 120°, and used for tangential and cross-sectional analyses in the sapwood rings 1989, 2002, and 2012.

### Sample processing and measurement of ray features

Wood sample where processed according to Schweingruber and Poschlod (2005) for the anatomical characterization and quantification of the rays. Therefore, 10–15-μm permanent thin sections were produced with a sledge microtome (Gärtner et al., 2014), placed on a slide and stained with Alcian blue (1% solution in acetic acid) and safranin (1% solution in ethanol) to differentiate between unlignified (blue) and lignified (red) cells. Afterwards, sections were dehydrated using a series of ethanol solutions of increasing concentrations, washed with xylol, and finally permanently preserved by embedding them into Eukitt glue (Gärtner and Schweingruber, 2013). Overlapping images covering the entire samples were captured with a Nikon D90 digital camera mounted on a Nikon Eclipse 50i optical microscope with 40 × magnification and merged using PTGUI v8.3.10 Pro (New House Internet Services B.V., Rotterdam, the Netherlands). In the (subset) of the 40 cores, the average measured width and surface in cross-sectional, tangential and radial sections were 6.69 mm, 4.87 and 9.39 mm^2^, respectively. In the stem disc the average measured width in cross-sections was 19 mm, while the average measured surface in tangential sections was 28 mm^2^.

The outlines of all rays and tree ring borders were vectorized manually in the images using a tailored version of ROXAS 1.6 (von Arx and Dietz, 2005; von Arx and Carrer, 2014), a specialized image analysis tool for wood cell anatomical measurements based on Image-Pro Plus (Media Cybernetics, Silver Spring, Maryland, USA). As a result ROXAS provided several statistics in a global and annual resolution such as individual ray area (all planes), individual ray length (cross- and radial sections) and ray height (tangential sections), the number and position of initiating (NEWRAY) and disappearing rays within the ring (cross-sections), and the percentage of ray surface in the xylem (PERPAR; all cutting planes).

### Study design

Several trials were used to identify reliable quantification methods and assess distinctness of ray metrics based on the subset of the six cores. First, the consistency of the measured percentage of ray surface in the xylem (PERPAR) in the three cutting planes (cross-, tangential, radial plane) was evaluated by comparing values from the same annual rings. Second, to explore the potential of more efficient ray quantification, the relationship of individual ray area and ray length (cross-sections) and ray height (tangential sections) was investigated by linear regression analyses. Third, the similarity of different ray parameters in the tangential sections such as PERPAR, ray density, ray area, ray height, and ray width was assessed by correlation analyses. Ray width was estimated from ray area through division by ray height.

The variability of ray parameters was assessed in two different ways. First, the inter-annual variability in cross-sectional PERPAR was determined in the full set of 40 cores and time series of 20 rings each by calculating the coefficient of variation (CV) and the mean sensitivity (Cook and Pederson, 2011), i.e., the average percentage change from one yearly value to the next. The same sample set was used to assess the intra-annual variability of cross-sectional PERPAR and the proportion of initiating rays (NEWRAY) between early- and latewood using *t*-test. Second, the variability of the obtained PERPAR values was assessed as a function of the measured wood surface by bootstrapping. Using tangential sections from each of the three locations in the three radial bars of the stem disc, one thousand 1-mm^2^ measurement windows were randomly placed and the PERPAR values were extracted and pooled to a single data pool (*n* = 9000). Bootstrapped values based on 1000 replications for PERPAR mean and coefficient of variation of its estimation (CV) were obtained by randomly combining an increasing number of individual 1-mm^2^ values from the data pool, thus simulating the measurement of increasing wood surface (from 1 to 15 mm^2^). The same procedure was repeated for the cross-sectional images from the same annual rings as the tangential sections for steps of 1-mm width (from 1 to 15 mm). Since mean ring width was around 1 mm (1.047 mm), a measured width of 1 mm in cross-sections corresponded to 1 mm^2^ in tangential sections, thus allowing direct comparisons of the obtained variability between the cutting planes. The calculated CVs were used to calculate the relationship between measured wood width/surface, number of samples (*n*), and estimated accuracy. These calculations were based on Equation (1):

(1) $$\begin{array}{r} CI95=2\cdot CV\cdot n^{-0.5} \end{array}$$

which expresses the relative 95% confidence interval (CI95; given as a percentage of the mean) as a function of CV and *n*. In fact CI95 approximately corresponds to twice the standard error (SE), SE equals to SD divided by the square root of the number of samples (*n*), and SD is CV multiplied by the mean.

## Results

**Figure 2:**
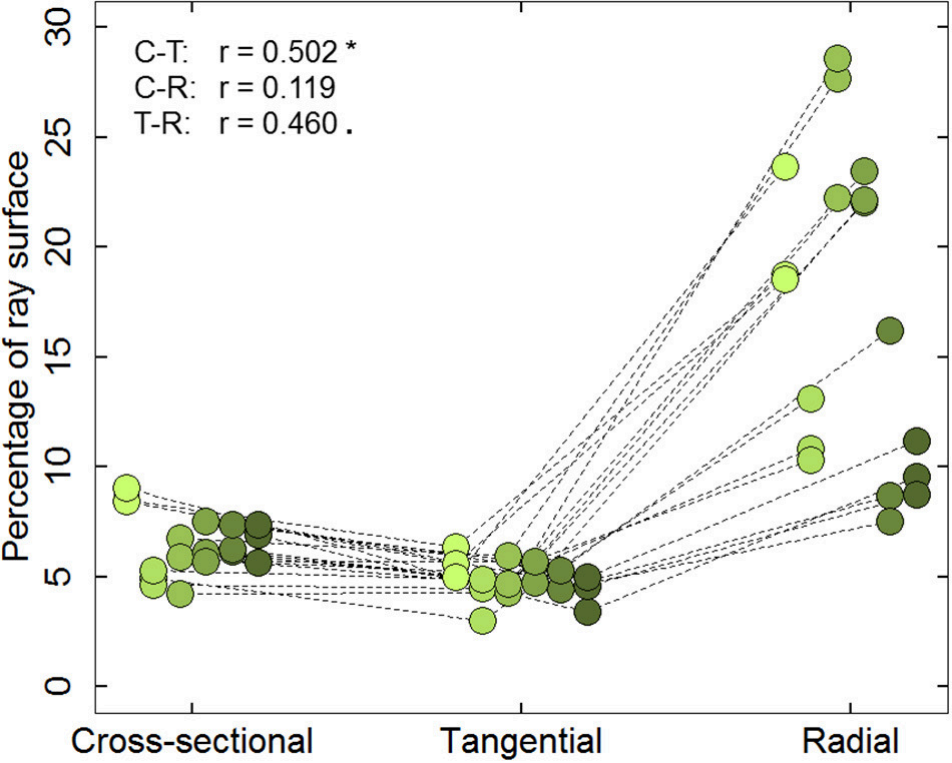
**Percentage of ray surface (PERPAR) measured on the cross-sectional, tangential and radial planes at three tree-rings (1989, 2002, and 2012) along the radial cores of six mature Scots pine trees**. Symbols of the same color represent the PERPAR values of a specific cutting. Dotted lines connect the measurements from the same tree and ring. Symbols of the same cutting plane are jittered along the x-axis for easier interpretation. Left upper data indicates the *Pearson* correlation coefficient (*r*) between cross- and tangential sections (C-T), cross- and radial sections (C-R), radial and tangential sections (R-T), respectively. ^*^P ≤ 0.05; · *P* ≤ 0.1.

Results from the six cores indicated that the percentage of ray surface (PERPAR) varied substantially depending on the cutting plane. However, PERPAR values within a cutting plane and core were rather similar (mean CV = 0.12, 0.16, and 0.17 for cross-, tangential and radial sections, respectively; Figure 2). Mean PERPAR value for cross-sections was 6.50%, which was significantly higher than for the tangential sections (4.92%; *t* = 5.606; *P* < 0.001). PERPAR was largest in the radial sections (16.84%), deviating significantly from the values of both other cutting planes (*t* = −6.287; *P* < 0.001 and *t* = −7.641; *P* < 0.001 for cross-sectional and tangential planes, respectively). Despite the differences, PERPAR values in cross- and tangential sections were significantly correlated (*r* = 0.502; *P* = 0.034).

**Figure 3:**
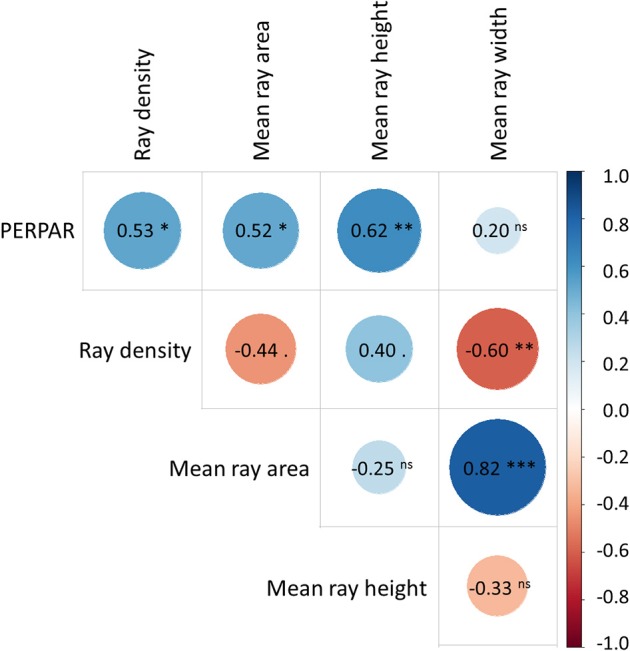
*****Pearson's*** correlation matrix between the ray parameters measured in tangential sections (***n*** = 18)**. The parameters include percentage of ray surface (PERPAR), ray density, mean ray area, mean ray height and mean ray width based on measurement from six mature Scots pine trees. For each tree, three locations separated by five to seven annual rings were analyzed. ^***^P ≤ 0.001; ^**^P ≤ 0.01; ^*^P ≤ 0.05; · *P* ≤ 0.1; ns, not significant.

The analysis of the subset of six cores showed that individual ray area was highly correlated with ray length in the cross-sectional (*n* = 2017; *R*^2^ = 0.926; *P* < 0.001) plane and ray height in the tangential plane (*n* = 13.218; *R*^2^ = 0.847; *P* < 0.001). In tangential sections correlation analyses revealed that PERPAR is significantly correlated with all other parameters except for mean ray width (*Pearsons's r* = 0.196, *P* = 0.435; Figure 3). At the section level we also observed that mean ray width was highly correlated to mean ray area (*r* = 0.824; *P* < 0.001), but was unrelated to mean ray height (*r* = −0.326, *P* = 0.186).

**Figure 4:**
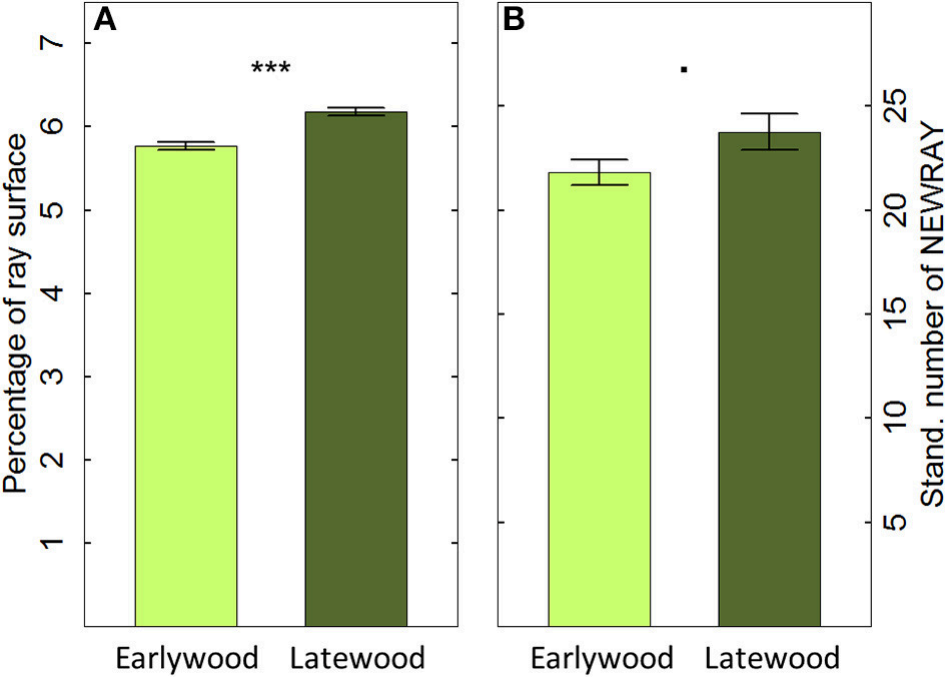
**Percentage of ray surface (PERPAR, A) and number of initiating rays (NEWRAY, B) in early- and latewood based on cross-sectional measurement of 20 tree rings in 40 mature Scots pine trees (mean ±1 se; ***n*** = 800)**. NEWRAY is expressed as counts per width unit (mm) to account for different widths of early- or latewood (Stand. number of NEWRAY). ^***^P ≤ 0.001; · *P* ≤ 0.1.

The 40 cores showed an overall mean CV in the annual PERPAR of 0.121 (ranging from 0.069 to 0.171 among individuals) and a mean sensitivity (MS) of 10.8% (ranging from 6.0 to 17.2%). Moreover, PERPAR in the cross-sectional plane was significantly smaller in the earlywood (5.77%) than in the latewood (6.18%; *t* = 6.139; *P* < 0.001; Figure 4A). Similarly, the number of initiating rays (NEWRAY) after correction for unequal tissue contributions to the overall ring area differed marginally between earlywood (21.82 rays/mm) and latewood (23.75 rays/mm; *t* = −1.810, *P* = 0.070; Figure 4B).

**Figure 5:**
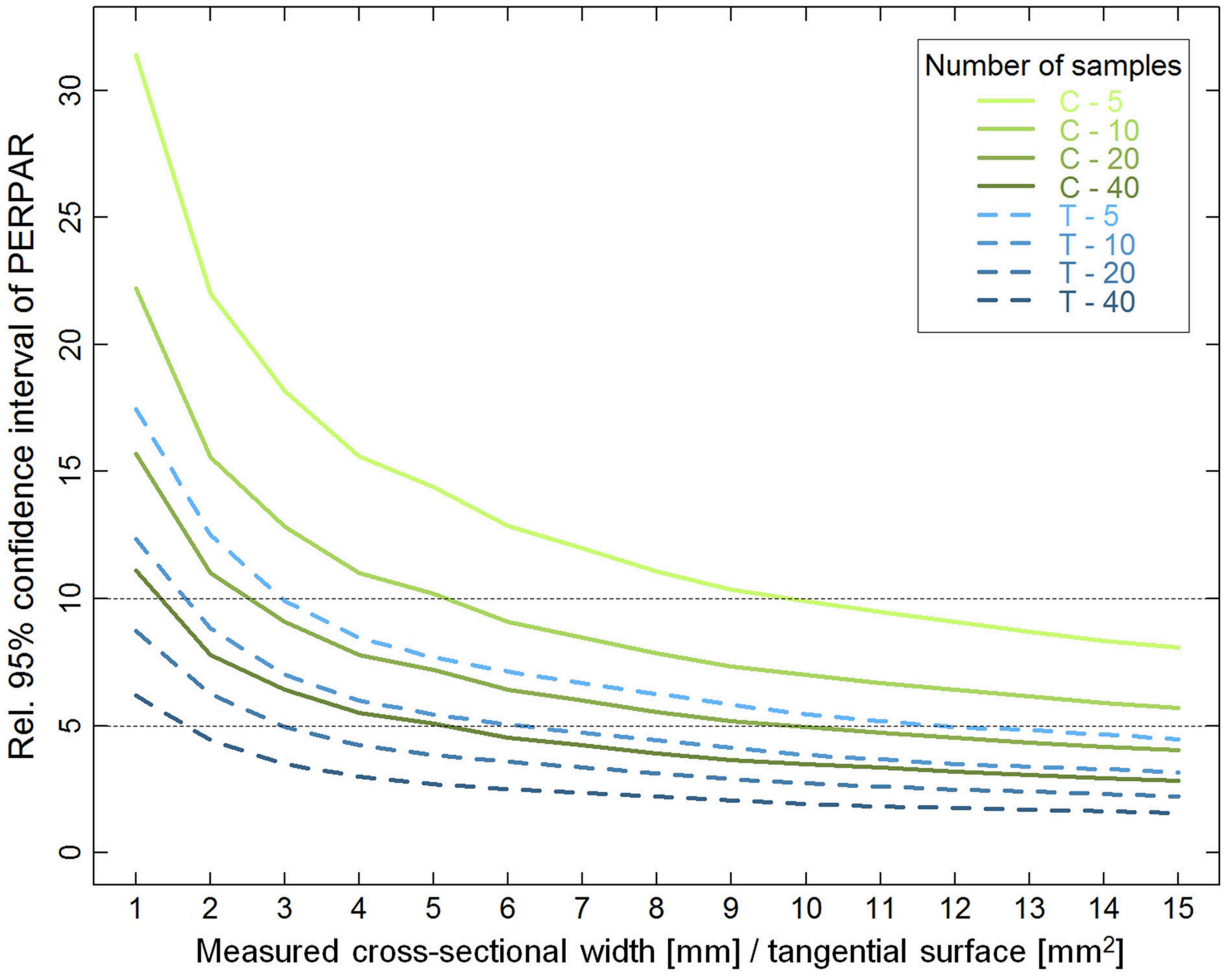
**Relative 95% confidence interval (CI95) of percentage of ray surface (PERPAR) as a function of measured wood width (cross-sections; C; green solid lines), surface (tangential sections; T; blue dashed lines), and number of samples**. The dotted horizontal lines delimit a band that might represent a reasonable balance between data accuracy and measurement efficiency. See Materials and Methods section for further explanations.

Bootstrapping analyses indicated that the variability of PERPAR mean estimation decreased with increasing measured wood width (cross-sections)/surface (tangential sections) and number of samples (Figure 5). However, the relative 95% confidence interval (CI95) was about twice as large in cross- than in tangential sections for a given measured wood surface. In cross-sections, CI95 curves flattened and got almost linear after a measured wood width of 8 mm, while this already occurred with 4–5 mm^2^ in tangential sections. Measurements from 10 cross-sections on 5-mm increment cores resulted in a CI95 of ±10.1% of the true mean PERPAR, doubling the number of samples to 20 reduced CI95 to ±7.0%, while increasing the measured width to 8-mm wide strips provided an accuracy of CI95 ±7.7% with 10 samples. In tangential sections, 10 of the 1-mm^2^ surfaces frequently used in previous studies (see Introduction) only provided a CI95 of ±12.7% of the true mean PERPAR. In contrast, measuring 2 × 2 mm areas in 10 samples provided a CI95 of ±6.0%, while it was reduced to even ±4.3% with twenty 2 × 2 mm samples.

## Discussion

### Estimates of percentage of ray surface depend on the cutting plane

In this study we evaluated several methodologies for quantifying ray features in conifers. Tangential and cross-sections proved to be suitable, but with strengths and limitations in terms of accuracy and investment, and with differences in the information they register. In contrast radial sections were generally unsuitable for ray quantification. The large differences in percentage of ray surface (PERPAR) estimation (up to three times) among cutting planes likely reflects sampling artifacts linked to the sheet-like orientation and fusiform shape of uniseriate rays (see Figures 1D,E). In fact, in a radial section, the position and contour of the ray outline may change substantially from the lower to the upper side of the section. Because of the transparency of the tissue, the perceived ray outline is the maximum ray projection through the entire thickness of the section, which leads to a systematic overestimation (“radial overestimation artifact”). This effect–although much weaker—also occurs in cross-sections due to the tapering toward the upper and lower ray extremities, supposedly explaining the overestimated values (Figure 2). In contrast, the tangential sections are robust in this respect and therefore likely produced most accurate estimates of PERPAR, and therefore relative ray volume (Myer, 1922). The significant correlation between tangential and cross-sectional data confirms the systematic nature of the larger cross-sectional values. With respect to the quantification of the area of individual rays, the strong allometric relationship allows accurate estimation of ray area as a function of ray length in cross-sections (Olano et al., 2013) and ray height in tangential sections. Using such relationships could significantly increase the efficiency of the measurement procedure.

### PERPAR and ray width are the most complementary tangential ray parameters

Among the different tangential ray parameters, PERPAR positively correlated with all other ray metrics but mean ray width, which suggests PERPAR incorporates the information from most other metrics (Figure 4). Notably, the significant correlations with mean ray area and height are in line with previously observed consistent patterns of these metrics in relation to tree vigor and growth conditions (e.g., Bannan, 1954, 1965; White and Robards, 1966; Fonti et al., 2015). Moreover, the missing relationship between mean ray height and width indicates that their ratio mainly varied among the analyzed images (i.e., trees and/or annual rings), since within the images individual ray height well scaled to the width (mean *Pearson's r* = 0.475; *P* < 0.001). This variability is mainly due to the variability in ray width: supplementary analyses revealed that the width of individual rays is less strongly correlated with ray area (mean *Pearson's r* = 0.750; *P* < 0.001) than ray height (mean *Pearson's r* = 0.899; *P* < 0.001). Mean ray width also showed a larger variability among trees and/or annual rings than mean ray height (CV = 0.169 vs. 0.094). Together with its independence from PERPAR, mean ray width might therefore be a more promising parameter for ecological studies than mean ray height.

### Spatial variability of ray features within tree rings

The higher PERPAR and marginally larger number of initiating rays (NEWRAY) observed in the latewood compared to earlywood was unexpected when considering the radial orientation of the rays. In fact, once initiated, rays grow and extend to keep the connection with the cambium and phloem (Fischer and Höll, 1992; Spicer, 2014). Such small intra-annual differences could indicate slightly larger parenchyma cells (DeSmidt, 1922) and/or a higher ray initiation rate in latewood than in earlywood. They potentially evidence an advantage of having a larger storage and transport capacity close to the cambium to support the onset of cambial activity in the following growing season. However, the small intra-annual difference in PERPAR values also indicates that it should suffice to only roughly balance early- and latewood in a proportional way when analyzing PERPAR in tangential sections. Additionally, if the section was not taken fully parallel to the orientation of the rays, the number of NEWRAY in cross-sections may be overestimated (see Figure 1B). The extent of overestimation in ray initiation is probably directly related to the number of disappearing rays (“ending ray artifact”; Eckstein, 2013), because they are both directly linked to the orientation of the section (see Figure 1C). Nevertheless, time series of the number of initiating rays from samples with an orientation problem still represent valid data if standardized before statistical analysis.

### Accuracy of PERPAR values depending on measurement width and area

The accuracy of PERPAR greatly changed depending on the measured wood width (cross-sections), surface (tangential sections), and the number of samples (Figure 5). Yet, our results suggest that common measurement strategies—10 cross-sections of 5-mm increment cores or 10 tangential planes of 1 mm^2^—provide rather inaccurate estimates of PERPAR. They may thus be often inappropriate to extract an ecological signal considering that year-to-year variability (MS) in PERPAR is about 20% (Olano et al., 2013; Fonti et al., 2015), or even only about 11% as observed in the 40 cores analyzed here. This study estimates how an increased number of samples [since, CI95 decreases with the square root of the number of samples (cf. Equation 1)], or expansion of the measured wood width/area will increase the accuracy. Although our assessment was based on a very detailed analysis of a single stem disc, it cannot be excluded that the CI95 curves may be shifted for Scots pines from other populations or different species. However, we speculate here, that a CI95 in the range between ±5 and ±10% (dotted horizontal lines in Figure 5), corresponding to approximately half the CV or MS, could represent a reasonable balance between data accuracy and measurement efficiency.

## Conclusions

Our results suggest that both cross- and tangential sections are suitable for quantitative approaches, whereas radial sections are generally unsuitable due to strong sampling artifacts. Table 2 summarizes the major potential and pitfalls recognized in this study. Our main conclusions are that the quantification of rays in cross-sections is generally very efficient and allows establishing annual time series of (consistently overestimated) ray volume and number of initiating rays that can be compared to other anatomical traits such as tracheid dimensions, cell wall thickness and resin ducts, and related to time series of environmental conditions. Tangential sections seem more suitable to accurately estimate ray volume and investigate the spatial integration of rays in the xylem such as the connectivity of conduits to rays, or, more generally, research into structure-function relationships. In addition, tangential ray width registers information independent from PERPAR, which makes it a promising complementary parameter for ecological studies. The choice between tangential and cross-sections will therefore depend on the specific study question and on the available lab capacities. In this context, this study presents for the first time a very concrete guidelines (Figure 5) for estimating data accuracy depending on the size of the measured wood width (cross-sections), surface (tangential sections), and number of samples, helping to define a suitable sampling strategy, although the latter also depends on the known or expected responsiveness and variability of the target ray features.

**Table 2:** **Potential and pitfalls of anatomical ray quantification in conifers for different cutting planes**.

	**Cross-section**	**Tangential section**	**Radial section**
Potential	- Efficient creation of annual time series - Clear assignment to early- vs. latewood - Comparison with other anatomical variables on same image (ring width, tracheid size, cell wall thickness, etc.)	- Most accurate estimate of relative ray volume - Good estimation of ray density in 3D and connectivity with conduits - Robust against deviations in cutting orientation - Commonly used 5-mm cores provide accurate results based on 4-mm^2^ measured areas and *n* = 10	- Inspection of cutting orientation in cross-sections to assess the quality of the number of initiating rays
Pitfalls	- Systematic overestimation of relative ray volume - Non-perpendicular cuttings: overestimation of number of initiating rays; permanently disappearing rays “ending ray artifact” - Transiently disappearing rays - Large sample size (*n* ≥ 20) required to obtain accurate results with commonly used 5-mm wide cores	- Time consuming when creating annual time series	- Substantial overestimation of ray surface (“radial overestimation artifact”) - Very unreliable for most of the parameters

Since, most conifer species display a similar ray architecture and a relatively narrow range of ray volume, we are confident that most of our results are representative for other conifer species as well. A similar assessment might be applied to identify the best methodology in angiosperm species. We are convinced that the methodological guidelines presented here are necessary to foster the establishment of robust quantifications, which will ultimately improve the understanding of the fundamental role of ray parenchyma tissue for the performance and survival of trees growing in stressed environments.

### Conflict of interest statement

The authors declare that the research was conducted in the absence of any commercial or financial relationships that could be construed as a potential conflict of interest.
